# Complications and benefits of enteral feeding in children with progressive neurological disease in a palliative care service: a retrospective study

**DOI:** 10.25122/jml-2024-0315

**Published:** 2024-09

**Authors:** Mihaela Hizanu, Mădălina Duceac, Letiția Doina Duceac

**Affiliations:** 1 Lumina Association - Bacau Palliative Care Center, Bacau, Romania; 2 Faculty of Medicine and Pharmacy, Dunarea de Jos University of Galati, Galati, Romania; 3 Prof. Dr. N.Oblu Clinical Emergency Hospital, Iasi, Romania

**Keywords:** palliative care, pediatrics, enteral feeding, need, neurological disease, life-limiting illness

## Abstract

Palliative care for children with neurological conditions is essential to improve their quality of life and that of their family, given their uncontrollable symptoms and associated disabilities. These conditions include genetic diseases, congenital brain malformations, neurodegenerative disorders, and acquired brain injury. Enteral feeding, given directly into the gastrointestinal tract, is often necessary. A retrospective study conducted between 2018 and 2023 at the Lumina Association - Bacau Palliative Care Center analyzed data from 604 children with progressive neurological disorders out of 952 patients. These children, with an average age of 9.03 years, required enteral feeding due to swallowing disorders (48%), congenital malformations (29%), and malnutrition (23%). Feeding was performed mainly through a nasogastric tube (97.52%) and in 2.48% of cases through a gastrostomy. During this period, 4.14% of patients died from the underlying disease. The study highlights the benefits and complications of enteral feeding in these children. Although enteral feeding has been shown to be effective in maintaining nutritional status and avoiding dehydration, challenges have been identified, including digestive complications and the risk of infections in the context of palliative care.

## INTRODUCTION

Patients in palliative care with progressive neurological conditions are the most common group requiring enteral feeding. Nutrition and symptom control are some of the most important aspects of their care [1]. Children with life-limiting illnesses and, therefore, with palliative care needs face difficulties with nutrition as their illness progresses. Thus, regular nutrition assessment is crucial, considering the family's psychosocial needs, the child's underlying condition, the stage of disease progression, treatment and associated complications, and the child's energy and nutritional needs [2]. The main aim is to ensure a good quality of life during the stages of palliative care, and the balance between the benefits and burden of any planned intervention must be carefully assessed according to the particularities of each child and his/her family. Addressing feeding issues in palliative care begins and progresses throughout the disease progression to the terminal stages of care, giving the family some control in such a difficult situation for their child [3].

Children with progressive neurological diseases have difficulty feeding and develop gastrointestinal symptoms, often leading to malnutrition. This results in decreased quality of life for patients and their caregivers. In general, quality of life is an individual's perception of everyday life, including all aspects of emotional, social, and physical life. In health care, quality of life refers to the assessment of how a person's well-being may be affected by an illness, disability, or disorder [4,5].

Caregivers of pediatric patients with progressive neurological disorders have been shown to have a very low quality of life, poor mental health, and higher levels of burnout, often developing burnout overuse syndrome [6-10]. Enteral tube feeding is increasingly used to improve the growth and nutritional status of children with oral dysphagia associated with progressive neurological disorders and undernutrition [11].

The 2017 European Society of Pediatric Gastroenterology, Hepatology, and Nutrition (ESPGHAN) guidelines recommend enteral nutrition as a safe and qualitative nutritional intervention for children with neurological conditions in palliative care. It has proven effective in combating malnutrition in this group of patients and positively impacts family routine and social dynamics. Enteral nutrition is usually initiated in hospitals, but many patients require continued enteral nutrition at home, thus avoiding high costs and complications or infections associated with hospitalization [12].

Enteral feeding is indicated in the case of insufficient oral intake (e.g., in heart disease or chronic respiratory disease) or swallowing difficulties with the inability to chew and swallow (e.g., in severe mucositis or neurological disorders). The type of enteral feeding is chosen according to clinical considerations and the preferences of the child and the family. Methods such as a nasogastric tube, oro-gastric tube, nasogastric-jejunal tube, percutaneous gastrostoma, or percutaneous jejunostoma are recommended. Specific pharmaceutical preparations are administered, and home-prepared pureed foods are not recommended as a first option unless the parents prefer this or the pharmaceuticals are unavailable. These foods can be fortified with macronutrients (carbohydrates, lipids, and proteins) available in powdered or liquid form that are mixed with the food, as well as micronutrients (vitamins, minerals, etc.) [13].

The objective of this study was to evaluate the impact of enteral nutrition on nutritional status, quality of life, and family dynamics in palliative care in children with progressive neurologic disorders. Specifically, the research aimed to identify the benefits and limitations of this intervention, to analyze the optimal ways to implement it in hospice palliative care units for children, and to evaluate the long-term effects on patients and their caregivers.

## Material and Methods

### Study design

We conducted a retrospective study from 2018 to 2023 involving children diagnosed with life-limiting diseases and neurological impairments. The study occurred at the Lumina Association - Palliative Care Center for Children in Bacau, a unique hospice facility in Romania offering continuous inpatient care.

### Inclusion and exclusion criteria

We included pediatric patients diagnosed with progressive neurologic disorders, aged 0-18 years, and who have been fed by nasogastric or gastrostomy tube.

We excluded children diagnosed with life-limiting diseases who did not require enteral feeding.

### Data collection

Several parameters were monitored: age, gender, diagnosis, presence or absence of O2 ventilation, recommendation for enteral feeding, mode of enteral nutrition (nasogastric tube or gastrostomy), and type of product used for enteral feeding. Our research aimed to identify the benefits and challenges encountered during care (adverse events, complications, or healthcare-associated infections) to improve the quality of life of these patients. Statistical-mathematical methods were used to interpret the results, and the frequencies of various variables were calculated.

## RESULTS

**Figure 1:**
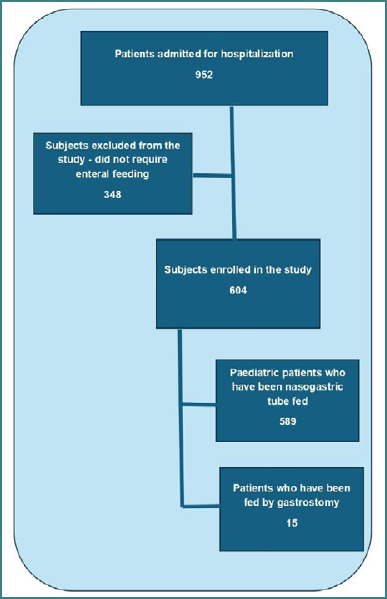
Flowchart of hospitalized children requiring enteral feeding

Our research included 952 patients who accessed our continuous hospitalization palliative care service (hospice type) between 2018 and 2023. These patients were admitted due to uncontrolled impairments and symptoms associated with a life-limiting illness. Of these, 348 (36.55%) were excluded from the study because they did not require enteral feeding. A total of 604 hospitalized children who required enteral feeding were enrolled in the study group. Of these, a total of 589 (97.52%) were fed by nasogastric tube and 15 children (2.48%) by gastrostomy (Figure 1).

**Figure 2:**
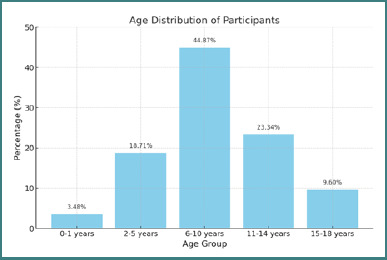
Age distribution of participants

The mean age of patients in the study group was 9.03 years. A significant number of patients, 21 (3.48%), were under one year old (Figure 2), indicating that neurological issues and the need for enteral feeding may occur very early in life. Boys were more affected than girls, with 461 boys (76.32%) compared to 142 girls (23.68%), resulting in a female-to-male ratio of 0.31.

The data in Figure 2 indicate that enteral feeding should be adapted to individual medical needs and the child's age. The age group with the highest representation in the study was 6-10 years (44.87%). Managing enteral feeding can be more complex in this age range due to greater individual differences in growth and development. Energy requirements increase during this period, and dietary needs become more diverse. Also, progressive neurological conditions may begin to significantly impact the ability to swallow or chew, making enteral feeding essential.

For the youngest age group (0-1 year), which accounted for 3.48% of the study population, enteral nutrition can be crucial for proper development and ensuring nutritional requirements in severe neurological disorders. Breastfeeding or specialized formula is commonly used, and caloric requirements are high to support rapid growth.

Regarding neurological disorders, the percentage distribution of diagnoses within the target group is shown in Table 1. The data revealed that the most common diagnosis was spastic tetraparesis, affecting 46.5% of the children (281 patients), followed by chronic infantile encephalopathy at 17.9% (108 patients) and cerebral palsy at 15.6% (94 patients). Less common conditions included paraplegia at 5.8% (35 patients), hydrocephalus at 4.6% (28 patients), and hemiplegia at 4.6% (28 patients). A smaller number of children were diagnosed with microcephaly (1.8% - 11 patients), spina bifida (1.3% - 8 patients), and partial cerebellar abnormalities (0.3% - 2 patients). The frequency analysis indicates that the most common diagnoses in the target group were spastic tetraparesis, followed by chronic infantile encephalopathy and cerebral palsy. The large number of children with spastic tetraparesis in the target group suggests that almost half of the patients had a severe form of paralysis involving all four limbs, indicating a significant need for nutritional support and specialized care.

**Table 1:** Diagnostic distribution of neurological conditions

Diagnostic condition	Frequency	Percent (%)
Spastic tetraparesis	281	46.5
Chronic childhood encephalopathy	108	17.9
Hydrocephalus	28	4.6
Paraplegia	35	5.8
Cerebral palsy	94	15.6
Corpus callosum agenesis	9	1.5
Partial cerebellar anomalies	2	0.3
Microcephaly	11	1.8
Spina bifida	8	1.3
Hemiplegia	28	4.6
Total	604	100.0

Regarding enteral feeding methods (Table 2), 97.52% of the children (589 patients) were fed using a nasogastric tube, while gastrostomy was used in only 2.48% (15 patients).

**Table 2:** Basic characteristics of the study group

Characteristic	Value
Age of hospice admission	Mean 9.03
Age < 1 year	21 (3,48%)
Sex (M:F)	462:142
Total	T=604
The enteral feeding route	94
Nasogastric tube	589 (97,52%)
Gastrostomy	15 (2,48%)
02 ventilation	169 (27,98)
Deaths	25 (4,14%)

**Figure 3:**
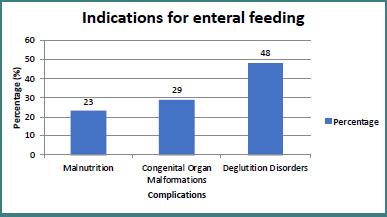
Indications for enteral feeding

The study also analyzed the indications for initiating enteral feeding. Nearly half (48%) of the hospitalized children had swallowing disorders that prevented safe oral feeding, emphasizing the critical need for enteral nutrition in these cases. Additionally, 29% of the children in the study group had congenital organ malformations (e.g., esophageal atresia, cleft palate, heart malformations). These malformations further complicate feeding and may require specialized nutritional solutions (Figure 3). For example, esophageal strictures or cleft palate could obstruct effective oral feeding, while cardiac malformations could lead to fatigue and breathing difficulties during feeding.

Another 23% of the children in the study had malnutrition due to prematurity and dysmaturity. Premature infants and those with dysmaturity (low birth weight for gestational age) have increased nutritional requirements and often have difficulty obtaining adequate oral nutritional intake (Figure 3).

The nutritional status of the children was evaluated at admission and then reassessed 6 months after the initiation of enteral feeding.

The initial mean nutritional status was 2.22, indicating that most children were at risk of malnutrition or already malnourished (Table 3). Six months later, the mean value had improved to 1.77, suggesting that most children were at risk of malnutrition or had achieved a normal nutritional status. The standard deviations (0.44 initially and 0.45 after 6 months) and small coefficients of variability (19.7% and 25.3%) indicated that the target group is homogeneous in terms of nutritional status both at baseline and at the 6-month reassessment.

**Table 3:** Descriptive statistics for nutritional status at initial assessment and re-evaluation at 6 months

Statistic	Nutritional status initial	Nutritional status re-evaluation at 6 months
*n*	604	604
Mean	2.22	1.77
Median	2.00	2.00
Mode	2.00	2.00
Standard Deviation	0.438	0.447
Coefficient of variation (%)	19.7	25.3
Minimum	1.00	1.00
Maximum	3.00	3.00

Initially, 76% of the subjects (459 children) were at risk of malnutrition, 23% (139 children) were malnourished, and only 1% (6 children) had a normal nutritional status (Table 4). After 6 months, the number of children with a normal nutritional status increased to 24.2% (146 children), the number at risk of malnutrition slightly decreased to 74.7% (451 children), and those with malnutrition dropped to 1.2% (7 children). The percentage analysis showed a 23.2% increase in children with normal nutritional status, a 1.3% reduction in those at risk of malnutrition, and a 21.8% decrease in malnourished children.

**Table 4:** Nutritional status at initial assessment and re-evaluation at 6 months

Nutritional status	n (Initial)	Percent (Initial)	n (6 Months)	Percent (6 Months)
Normal nutritional status	6	1.0	146	24.2
Risk of malnutrition	459	76.0	451	74.7
Malnutrition	139	23.0	7	1.2
Total	604	100.0	604	100.0

Various types of enteral formulas were used to correct malnutrition in the children included in the study, adapted to the nutritional and clinical needs of each individual. The formulas included standard, hydrolyzed, and semi-elemental variants, chosen based on individual tolerance and pre-existing gastrointestinal symptoms.

Table 5 shows the percentage distribution of different milk formulas in the target group. We observed that 11.4% (69 patients) used Nutren junior, 12.1% (73 patients) were fed Nutrison powder, 8.8% (53 patients) were fed resource protein + blended food, 8.8% (53 patients) were fed Resource protein + blended food, 36.6% (221 patients) were fed special milk formula + blended food, and 22.4% (135 patients) were fed only blended food. The percentage frequency analysis shows that the majority of subjects were fed with special milk formula + blended food and blended food alone.

**Table 5:** Distribution of special milk formulas used in the study group

Special milk formula	Frequency	Valid Percent (%)
Nutren Junior	69	11.4
Nutrison Powder	73	12.1
Polycal - Nutricia	53	8.8
Resource protein + blended food	53	8.8
Special milk formula + blended food	221	36.6
Blended food	135	22.4
Total	604	100.0

Another result obtained in our study was the effect of the special milk formula used, the diagnosis, and the testing phases on the nutritional status. ANOVA test was used to see if the special milk formula, the diagnosis, and the evaluation phases have individual and/or combined effects (through interactions) on the nutritional status of the patients (Table 6).

**Table 6:** Effect of milk formula used, diagnosis, and testing phases on nutritional status

Tests of Between-Subjects Effects								
Dependent Variable: Nutritional_status								
Source	Type III Sum of Squares	df	Mean Square	F	Mr.	Partial Eta Squared	Noncent. Parameter	Observed Power^b^
Corrected Model	96.304^a^	95	1.014	4.002	.000	.255	380.178	1.000
Intercept	726.396	1	726.396	2867.593	.000	.721	2867.593	1.000
Special_milk_formula	2.602	5	.520	2.054	.069	.009	10.272	.689
Diagnostics	1.436	9	.160	.630	.772	.005	5.667	.317
Stages_of_evaluation	13.854	1	13.854	54.691	.000	.047	54.691	1.000
Special_milk_formula * Diagnosis * Stages_of_evaluation	32.205	80	.403	1.589	.001	.103	127.136	1.000
Error	281.683	1112	.253					
Total	5226.000	1208						
Corrected Total	377.987	1207						

a. R Squared = .255 (Adjusted R Squared = .191); b. Computed using alpha = .05

The two-way ANOVA results in Table 6 reveal several key findings regarding the factors influencing nutritional status. First, the analysis showed no significant main effect for the type of special milk formula used [F (5,1112) = 2.05, *P* = 0.069, partial η^2^ = 0.009], indicating that the milk formula did not significantly affect nutritional status, as the *P* value was slightly above the 0.05 threshold. The partial eta squared value of 0.009 suggests that only 0.9% of the variance in nutritional status could be attributed to the milk formula, representing a very small effect size.

Similarly, there was no significant main effect for diagnosis [F (9,1112) = 0.63, *P* = 0.772, partial η^2^ = 0.005], suggesting that the specific diagnosis had minimal influence on nutritional status. The partial eta squared of 0.005 indicated that a mere 0.5% of the variance could be explained by the diagnosis, an extremely small effect.

Conversely, the assessment stages (baseline vs. 6-month reassessment) had a significant impact on nutritional status [F (1, 1112) = 54.69, *P* < 0.005, partial η^2^ = 0.047]. The *P* value below 0.005 indicated a statistically significant effect, with the partial eta squared showing that approximately 4.7% of the variation in nutritional status was explained by the changes between the two-time points, a moderate effect size. Furthermore, there was a significant interaction between the special milk formula, diagnosis, and assessment stages [F (80, 1112) = 1.59, *P* = 0.001, partial η^2^ = 0.103]. This interaction suggests that the combined influence of these factors significantly affected nutritional status, as indicated by the *P* value of 0.001. The partial eta squared of 0.103 indicates that 10.3% of the variation in nutritional status could be attributed to the interaction of these variables, representing a relatively large effect. The interpretation of these findings, specifically regarding complications of enteral feeding in children with progressive neurological diseases in a palliative care setting, reveals several important aspects (Table 7).

**Table 7:** Complications associated with the use of nasogastric and gastrostomy tubes for enteral feeding

Complications	*n* (%)
**Nasogastric tube complications (*n* = 589)**	
Nasal irritation and ulceration	84 (14.26%)
Aspiration of gastric contents	2 (0.34%)
Nasogastric tube blockages	17 (2.89%)
Gastrointestinal complications	65 (11.04%)
Dislocation or accidental removal of the tube	178 (30.22%)
Medication-related complications (catheter blockage)	77 (13.07%)
No complications	166 (28.18%)
**Gastrostomy complications (*n* = 15)**	
Infection at the stoma site	4 (26.67%)
Skin irritation and ulceration	5 (33.33%)
Accidental administration of food into the gastrostomy balloon	1 (6.67%)
Tube obstruction	4 (26.66%)
Accidental dislodgement or removal of the tube	1 (6.67%)
No complications	0

Several complications were noted in the study group of children using nasogastric tubes (*n* = 589). The most common was dislocation or accidental removal of the tube (30.22%), often requiring reinsertion and posing risks of discomfort and infection. Children with severe neurological disease may have muscle spasms, involuntary movements, or may be agitated, which increases the risk of accidental tube removal.

Nasal irritation and ulceration were also frequent (14.26%), indicating discomfort from long-term tube use. Medication-related complications, such as catheter blockage (13.07%), highlighted the challenges of administering medications through the tube. Some medications do not completely dissolve in water or other solutions, which can lead to the formation of precipitates that clog the tube.

Gastrointestinal complications (11.04%) indicate digestive problems related to administering nutrient formulas, such as abdominal bloating, diarrhea, or constipation. Abdominal distension can be caused by the accumulation of gas, fluid, or inadequately digested food. Nutritious formulas, although essential to meet caloric and nutritional needs, can cause digestive problems if they are not well tolerated by the child. Even feeding too quickly or in too large volumes can lead to abdominal distension and discomfort.

Nasogastric tube blockages in children with severe neurological conditions in palliative care were relatively rare (2.89%) but significant complications. These blockages can cause abdominal discomfort, interrupt enteral feeding, and increase the risk of aspiration. Appropriate management of medication and nutrient formula administration, as well as regular tube flushing, are essential to prevent and minimize these complications. Although rare, aspiration of gastric contents (0.34%) was a serious complication that can lead to aspiration pneumonia and other severe respiratory complications. Nearly one-third of the children (28.18%) in the study group did not experience complications, suggesting that enteral feeding by nasogastric tube can be administered effectively and safely under certain conditions.

Among children with gastrostomy tubes, the most common issues were skin irritations and ulcerations (33.33%) and stoma site infections (26.67%). Tube obstructions (26.66%) and accidental food administration into the gastrostomy balloon (6.67%) were less common but required attention and prompt intervention. Although rare, accidental tube dislocation (6.67%) may require immediate medical intervention (Table 7). Effective management of these complications requires careful monitoring, adequate training of staff and caregivers, and prompt, safe, and appropriate interventions.

## Discussion

The benefits of enteral nutrition in children with progressive neurologic disorders in the context of life-limiting illness include better weight gain, improved nutritional status, ease of feeding, improved medication compliance, and symptom control [14,15].

Enteral nutrition is recommended for patients with a functional gastrointestinal tract who are unable to orally consume sufficient nutrients to meet their estimated needs. The choice of the appropriate enteral access device depends on the patient's gastrointestinal anatomy and function, the anticipated duration of enteral nutrition, and the risk of aspiration. The nasoenteral tube is the most used enteral access method because it can be inserted into the stomach, duodenum, or jejunum. These tubes are intended for short-term use (less than 4 weeks) due to low complication rates, low cost, and ease of placement. Gastrostomies are indicated for long-term feeding (more than 30 days) or when obstructions make nasal intubation impossible [16].

Our study indicates that a significant proportion of children in the study group received enteral tube feeding, highlighting it as the predominant method used. This preference could be attributed to factors such as the simplicity of insertion, lower cost, and greater flexibility in usage. On the other hand, the low number of gastrostomy patients indicates that, although this method may offer long-term benefits and increased comfort for some patients, it is less commonly used. Possible reasons include the need for surgical intervention for fitting, the risks associated with the procedure, as well as prognostic and life expectancy considerations.

In this context, the nasogastric tube is preferred due to its practical advantages and less invasive nature, making it a more convenient choice. However, gastrostomy remains an appropriate option in cases where it is medically justified and accepted by the patient and family.

The European Society of Pediatric Gastroenterology, Hepatology, and Nutrition (ESPGHAN) 2017 guidelines [17,18] recommend choosing enteral nutrition as a safe and quality nutritional intervention for children with neurological conditions. It has been shown to be effective in combating malnutrition in this group of patients and has a positive impact on family routine and social dynamics. Enteral nutrition is usually initiated in hospitals and continued in all childcare settings.

According to the same ESPGHAN guidelines [17,18], gastrostomy is preferred when enteral feeding is required for more than 2 months because of its efficiency and increased patient comfort. Gastrostomy reduces the risk of complications associated with prolonged use of nasogastric tubes, such as nasal ulceration [19], which is common in children with progressive neurologic disorders, as was seen in our study. Despite clear recommendations for gastrostomy, the decision to switch to this method was postponed or not considered for various reasons: family preferences, fears about surgery (risk of death of the child), and financial and logistic considerations.

Other studies in a small group of children with severe psychomotor disabilities have shown that adequate nutritional support through less invasive enteral methods and better tolerated enteral formulas can improve weight, muscle mass, peripheral circulation, healing of decubitus ulcers, and general well-being while reducing irritability and spasticity [20,21].

Combination diets, which include commercial enteral formula and pasteurized foods prepared at the health facility, were used in the majority of cases in our study group. This approach has been effective in improving gastrointestinal symptoms and customizing nutrition according to the child's preferences and needs [22].

In the present study, we observed a higher number of male children compared to female children, which may suggest a genetic predisposition or other unidentified factors that make boys more susceptible to these conditions or lead to more frequent hospitalizations for symptom management. Additionally, with a significant portion of our patient population being infants and young children, there is a pressing need to develop and adapt specific techniques and protocols tailored for this age group. Notably, the majority of children hospitalized in our unit (63.45%) experienced oral feeding difficulties that necessitated enteral feeding. This high prevalence likely reflects the fact that our study group consists of severely disabled children requiring palliative care.

We observed that spastic tetraparesis and chronic childhood encephalopathy (CCI) were the most prevalent conditions among the children in our study. In this context, it is essential to tailor nutritional interventions and palliative care to address the specific needs associated with these conditions. These severe disorders impact motor function and swallowing, making enteral feeding necessary for adequate nutritional intake.

Swallowing disorders are common in children with progressive neurological diseases and can lead to severe risks of aspiration and malnutrition if not properly managed. Implementing enteral feeding, such as by nasogastric tube or gastrostomy, is essential to ensure adequate nutritional intake and prevent complications associated with inadequate oral feeding. Consistent with other studies, we found a strong relationship between the presence of feeding problems and the severity of disability [23]. Another study showed that feeding problems were specifically associated with reduced communication and manual skills [24].

Our results indicate that enteral feeding is essential to ensure adequate nutrition among these children, preventing malnutrition and supporting their growth and development despite severe medical conditions.

Assessment of malnutrition in children diagnosed with progressive neurological conditions in our study group was essential to ensure appropriate and personalized care. Given the complexity of these conditions and their impact on feeding and digestion, each child was evaluated for malnutrition and risk of malnutrition upon admission using the MNA-SF (Mini Nutritional Assessment - Short Form) score [25]. The MNA-SF is a shortened version of the Mini Nutritional Assessment tool, initially designed for evaluating malnutrition risk in elderly patients. However, it has been adapted for use in other populations, including children with chronic conditions or hospitalized [26]. Enteral feeding has also been shown to reduce episodes of pulmonary aspiration and other respiratory complications common in children with swallowing difficulties.

Enteral feeding may be vital to ensure the necessary intake of essential nutrients for these children, helping to correct nutritional deficiencies and improve quality of life. In addition, we observed that enteral feeding improved the overall health and quality of life of the children in the study.

Parents and caregivers reported a decrease in feeding-related stress levels, as enteral feeding provides a constant and controlled nutritional intake, which is difficult to achieve with oral feeding in children with severe disabilities.

One of the methods used for enteral feeding children in the study group was bolus feeding. This involved introducing a volume of food in a short time, usually between 5 and 30 minutes, using a syringe. This technique is similar to natural feeding, which may make the process more acceptable to children, with a similar rhythm and consistency to normal meals.

Implementing enteral feeding in children diagnosed with neurological diseases in palliative care brings with it a number of challenges. These can include the difficulty of administering enteral feeding, technically and in terms of coordination within the medical team. It can also be difficult to determine the right dose and type of feeding for each child, considering their needs and health status. The challenge is all the greater in children with progressive neurological diseases, as they may have swallowing difficulties or other problems that affect the feeding process [27,28].

Enteral feeding can be associated with certain complications and risks for children with swallowing difficulties. These include healthcare-associated infections, such as some associated with the feeding tube, or others, such as urinary tract infections, respiratory or aspiration infections, sepsis, or even local infections at the site of tube insertion [29-31].

In our study, 26.67% of children with gastrostomy had infection as a complication. This is a common complication that requires rigorous hygiene and medical supervision for prevention and treatment. The infection was treated with antibiotics, and proper stoma hygiene was maintained. Prevention involved regular cleaning and aseptic techniques while handling the gastrostomy. Irritation and ulceration were caused by gastric fluid leakage and friction. Protective skin barriers and protective creams were used to prevent their occurrence. This can occur at any time after placement. Most infections are minor and are usually manifested by tenderness, erythema, and purulent drainage at the stoma site [32]. Treatment of peristomal infections with antibiotics, administered through the tube, is usually successful, and the use of intravenous antibiotics is rare. Tube removal is rarely necessary to facilitate infection control. Predisposing risk factors for peristomal infection include underlying immunosuppression or tube traction [33].

Another important finding of our study was that a significant majority (72.52%) of the children experienced complications related to either nasogastric tube or gastrostomy use, while a smaller proportion (27.48%) did not encounter such issues. These results have key implications for medical practice:

- Need for preventive measures: Given the high rate of complications, it is imperative to develop and implement effective strategies to prevent complications associated with the use of the nasogastric tube and gastrostomy.
- Training of medical staff: Ensuring that medical staff are adequately trained in using and monitoring these devices is crucial. Continuous training and updating of medical knowledge can play a significant role in reducing complication rates.
- Close monitoring and follow-up: Regular and thorough monitoring of patients using nasogastric tubes or gastrostomies is necessary for the early detection and prompt management of complications.

We acknowledge several limitations in our study. First, as an observational, retrospective, single-center study, the findings may be influenced by confounding variables related to the recruited cases and clinical management practices specific to our center. Additionally, being a retrospective study, the analysis is limited to the data available and the clinical management approaches used during the study period. Consequently, our study and discussions do not claim to be a clinical guideline, nor do they represent a consensus for the nutritional management of enteral-fed children with progressive neurologic disorders.

### Relevance for future research and improving palliative care

The study suggests an urgent need for further research to optimize enteral feeding according to the specific type of neurological condition. This could include the development of personalized nutritional protocols and delivery techniques.

## Conclusion

Our study highlights that, despite potential complications, enteral feeding remains a valuable option for children with progressive neurological conditions in palliative care. Its benefits in providing adequate nutrition and reducing respiratory complications are significant, but a careful and multidisciplinary approach is essential for effectively managing complications. Although common complications such as catheter dislocation and nasal irritation have been identified, almost one-third of patients had no major problems. Gastrostomy use, although rare, was associated with benefits in some cases but required careful monitoring to prevent infection. These results emphasize the importance of adapting feeding plans and effective management of complications to improve the quality of life of these vulnerable children. The nutritional status of patients in the target group improved significantly 6 months after the initiation of enteral feeding. This improvement was reflected by weight gain appropriate to age and nutritional needs, stabilization of essential micronutrient levels, and reduced symptoms associated with malnutrition, such as muscle weakness, chronic fatigue, and frequent infections. Enteral feeding also helped to improve digestive tolerance and reduce episodes of gastro-oesophageal reflux and constipation, thus providing a constant and balanced nutrient intake personalized to each patient's needs. These positive results directly impacted overall health, increasing quality of life and reducing the need for additional medical interventions.

Further research and improved care protocols are needed to maximize the benefits of enteral feeding and minimize associated risks. The various neurological conditions, each with its complications and needs, underscore the need for individualized care plans. Each type of condition may present different challenges in the administration of enteral feedings, from the risk of aspiration to technical difficulties in the use of equipment. Our unit needs to implement some monitoring and maintenance procedures to prevent enteral feeding tube blockage.
